# Effects of Hand Hygiene Campaigns on Incidence of Laboratory-confirmed Influenza and Absenteeism in Schoolchildren, Cairo, Egypt

**DOI:** 10.3201/eid1704.101353

**Published:** 2011-04

**Authors:** Maha Talaat, Salma Afifi, Erica Dueger, Nagwa El-Ashry, Anthony Marfin, Amr Kandeel, Emad Mohareb, Nasr El-Sayed

**Affiliations:** Author affiliations: US Naval Medical Research Unit No. 3 (NAMRU-3), Cairo, Egypt (M. Talaat, S. Afifi, E. Dueger, A. Marfin, E. Mohareb);; Centers for Disease Control and Prevention, Atlanta, Georgia, USA (E. Dueger, A. Marfin);; Ministry of Health, Cairo (N. El-Ashry, A. Kandeel, N. El-Sayed)

**Keywords:** Hand hygiene, campaigns, influenza, viruses, schoolchildren, Egypt, absenteeism, expedited, research

## Abstract

To evaluate the effectiveness of an intensive hand hygiene campaign on reducing absenteeism caused by influenza-like illness (ILI), diarrhea, conjunctivitis, and laboratory-confirmed influenza, we conducted a randomized control trial in 60 elementary schools in Cairo, Egypt. Children in the intervention schools were required to wash hands twice each day, and health messages were provided through entertainment activities. Data were collected on student absenteeism and reasons for illness. School nurses collected nasal swabs from students with ILI, which were tested by using a qualitative diagnostic test for influenza A and B. Compared with results for the control group, in the intervention group, overall absences caused by ILI, diarrhea, conjunctivitis, and laboratory-confirmed influenza were reduced by 40%, 30%, 67%, and 50%, respectively (p<0.0001 for each illness). An intensive hand hygiene campaign was effective in reducing absenteeism caused by these illnesses.

Acute respiratory infections (ARIs) and diarrheal diseases cause substantial illness and death worldwide. Most of the estimated 5.5 million deaths associated with ARI and diarrhea occur in children from resource-limited countries. In these settings, where access to health services is often lacking, effective prevention methods are paramount. ARIs cause >4 million deaths annually and account for >7% of global deaths (*1*). Many ARIs are caused by viruses, including influenza A and B. Influenza viruses circulate in Egypt all year, peaking in winter months (*2*). Although influenza is generally self-limiting, each year it causes 3–5 million cases of severe illness and up to 500,000 deaths worldwide (*3*). The greatest number of excess deaths occur in persons >65 years of age, yet influenza greatly affects schoolage children as well. In addition, schoolchildren play a key role in transmission of influenza during community epidemics (*4*). Slowing or preventing transmission of influenza viruses among children may diminish the explosive transmission pattern that often characterizes annual influenza epidemics (*5*).

Diarrheal disease is the second leading cause of childhood illness and death and is responsible for ≈2 million deaths annually in children <5 years of age (*6*,*7*). Frequent and prolonged episodes of nonfatal diarrhea can lead to malnutrition, stunting of growth, and absenteeism in schoolage children (*8*,*9*). In Egypt, where child mortality rates have been reduced in recent years, diarrheal diseases still account for 13.9% of deaths in children <5 years of age. Much of the reduction in deaths caused by diarrheal diseases has resulted from better case management, including use of oral rehydration therapy and improved water and sanitation. Although deaths have decreased, the incidence of diarrheal diseases has remained relatively unchanged (*10*).

Hand hygiene is a key intervention for reducing transmission of ARI and diarrhea in community settings. Hand hygiene, using antibacterial soap or alcohol-based sanitizers, has been reported to result in notable reductions in the incidence of diarrheal diseases (*11*). Hand hygiene has also been specifically recommended for prevention of diseases with pandemic potential, such as severe acute respiratory syndrome and for influenza A pandemic (H1N1) 2009 (*12*–*14*). The objectives of this study were to measure the effectiveness of an intensive hand hygiene intervention campaign in reducing the incidence of absenteeism caused by illness and the incidence of laboratory-confirmed influenza in schoolchildren in Egypt.

## Methods

### Design

We conducted a randomized controlled trial to assess the effectiveness of an intensive hand hygiene campaign in reducing the absenteeism of schoolchildren due to illness, student in-class reported illness, and laboratory-confirmed influenza. The primary outcome measure was a determination of the rates of absenteeism caused by influenza-like illness (ILI) and laboratory-confirmed influenza, in which absenteeism caused by diarrhea and conjunctivitis were considered secondary outcomes. The study was performed over a 12-week period, February 16–May 12, 2008.

### School Settings

Cairo Governorate was chosen because of the continuous availability of water in school settings. The socioeconomic characteristics of families sending children to government schools in Cairo are homogenous in terms of education, income, and home sanitation facilities (*15*). Most schools in Cairo have 1 large restroom with ≈10 sinks and an additional 8–10 sinks on the playground. No sinks are available in the classrooms. Average size of each classroom is ≈48 m^2^ with 69 students (≈0.7 m^2^/student). Before the campaign, neither soap nor hand-drying material was available in the schools. Handwashing, if done at all, was only performed by rinsing hands in water. Hands were typically dried on clothing or air-dried.

### Sample Size

The sample size was calculated with the intent to detect a 20% reduction in laboratory-confirmed influenza in the intervention group, using a rate of 1.5% of laboratory-confirmed influenza per week in the control group and 70% participation. After doubling the sample to adjust for clustering, a total of 27 schools per group were adequate to detect this difference in laboratory-confirmed influenza with 80% power and 95% confidence. (The formula used to estimate the sample size is available at http://www.openepi.com/Menu/OpenEpiMenu.htm.) Sixty elementary schools (30 intervention and 30 control schools) were randomly selected from a numbered list of all 725 government elementary schools in Cairo by using a computer-generated random number table. All children at the intervention schools, regardless of grade, were included in the hand hygiene campaign activities, but absenteeism and illness data were only collected from children in the first 3 primary grades.

### School Teams

At each intervention school, a hand hygiene team composed of 3 teachers (social studies, arts, sports) and the school nurse was established. The hand hygiene team ensured that all predesigned activities for the hand hygiene campaign were implemented on a weekly basis (Table 1). The school nurses and teachers were trained to interview students, collect absenteeism data, interview parents, and complete data collection forms, and the nurses were trained to collect and process nasal swabs to test for influenza. At control schools, the nurses were supported by a single surveillance officer who was assigned to complete data collection forms.

**Table 1:** Hand hygiene campaign activities in intervention schools, Cairo, Egypt, February 16–May 12, 2008

Exercises, by week	School-based activities	Specific school initiatives
1. Game to explain germ theory	Obligatory handwashing twice daily for students under supervision	Handwashing champion of the week
2. How to wash hands	Soap made available	Theater play
3. Puzzle on handwashing	Morning broadcast	Best article contest on hand hygiene
4. Drawing portrait of person covering nose and mouth	Handwashing songs	Best speech contest on hand hygiene
5. Drawing with soap bars	Parents’ school meeting	Best drawing contest on hand hygiene
6. Soap bubbles game	Students-parents information transfer	Best singer contest
7. When to wash hands (open discussion)	Morning aerobics using handwashing songs	Best school contest
8. How to escape from the germs (game)		Formulation of handwashing committee
9. Germs transmission (game)		School trip to a soap plant
10. Where do germs live? (experiment)		School trip to a water purification plant
11. How sneezing contaminates hands (game)		
12. Germs characters (game)		

Although the methods for providing soap varied among the intervention schools, parents usually sent children to school with a small bag containing bar soap and a clean towel. If families could not afford soap and hand-drying material, the school administration provided them.

**S**ix independent social workers visited the schools weekly; each visited 10 schools per week (5 intervention and 5 control schools). During each visit, they observed hand hygiene activities, soap and drying material availability, and the process of students washing their hands during the school day; they also verified the accuracy of the illness data collected by teachers.

### Intervention Communication Campaign

An intensive campaign to promote hand hygiene was launched in the intervention schools to raise the awareness of students, teachers, nurses, and parents regarding the importance of hand hygiene and to increase the proportion of students washing their hands. Hand hygiene teams required students in the first 3 primary grades to wash their hands at least twice during the school day for ≈45 seconds, followed by proper rinsing and drying with a clean cloth towel.

Campaign materials were developed for 3 groups (students, teachers, parents). The teachers’ guidebook included a detailed description of the students’ activities and methods to encourage students to practice these activities. Posters were placed near sinks in the classrooms and on the playground. The primary message was to wash hands with soap and water upon arriving at school, before and after meals, after using the bathroom, and after coughing or sneezing.

Grade-specific student booklets were developed; each included a set of 12 games and fun activities that promoted handwashing. At least 1 activity was used each week. A special song to promote hand hygiene was developed and played regularly at schools. Informational fliers were distributed to parents to reinforce the messages delivered at the schools. Many schools were creative in motivating students to comply with washing hands, such as selecting a weekly hand hygiene champion, developing theater plays, and launching school contests for drawings and songs.

### Data Collection and Illness Definitions

Data were collected for 12 weeks during February–May 2008. The regulations of the Ministry of Education require schools in Egypt to record absences each day in a school log book, classified as absence caused by either an illness or a non-illness. The hand hygiene teams visited each classroom to verify the information collected by the school administration. They also telephoned parents of children absent due to illness on the first day of absence and interviewed them to complete an absenteeism data collection form that included specific symptoms of illness. Symptoms and signs of illness and detailed case definitions were the same as those used by Bowen et al. (*16*).

A student episode of absence caused by illness was defined as a student who was absent for any number of consecutive or nonconsecutive days during 1 calendar week with symptoms affecting the same organ system. The incidence of absence due to illness was defined as the number of absences caused by illness per 100 student-weeks. Rates of absence caused by illness with specific symptoms or signs were calculated as the number of absences due to illness associated with specific symptoms per 100 student-weeks. The incidence of in-class illness was defined as the number of reported in-class episodes of illness among the first 3 primary grade students per 100 student-weeks.

Children who were absent from school because of ILI (defined as fever >38°C and either cough or sore throat) were approached by the school nurse, who either visited the child at home (if possible) or asked the child to visit the school clinic if they returned to school within 3 days of absence. In addition, students who became ill during the school day were referred by teachers to the school clinic nurse. The school nurse interviewed the students and completed the in-class illness data collection form that included the same symptoms and signs used on the absenteeism data collection tool.

### Laboratory Methods

Nurses collected a nasal swab from children who visited the school clinic with ILI. Nasal swabs were collected by inserting and rotating a sterile swab into the anterior nares; the specimen was then tested for influenza A and B viruses by using QuickVue, a rapid, point-of-care antigen detection test designed for use by nonlaboratory personnel (QuickVue; Quidel Corp., San Diego, CA, USA). This test was conducted only for students who had prior written approval of a parent.

### Ethics and Informed Consent

The study protocol was reviewed and approved by the US Naval Medical Research Unit No. 3 (NAMRU3) Institutional Review Board (Protocol #NAMRU3.NAMRU3.2007–0007). A written letter describing the purpose of the study signed by the school principal of each school was distributed to the parents or guardians of all students in the first 3 primary grades. In addition, consent for obtaining a nasal swab from students reporting ILI was sought. Control schools received the same intervention program at the end of the study.

### Statistical Methods

To adjust for the cluster design effect, we calculated the rates of absenteeism and illness separately for each school. This was performed by dividing the number of episodes of absenteeism or illness by the number of student-weeks. The answer was then multiplied by 100 to obtain rates per 100 student-weeks. Since the rates were not normally distributed, the medians of the mean rates for the intervention and control schools were compared by using the Wilcoxon rank-sum test; p values <0.05 were considered significant.

## Results

During the 12-week observation period, 20,882 students (282,832 student-weeks of observation) were enrolled at the intervention schools in the first through third grades; 23,569 students (250,584 student-weeks) were enrolled at the 30 control schools. All parents approved providing information about their children’s illnesses; however, 7,112 parents (16%) did not give permission for the collection of nasal swab specimens from their children, and the distribution was similar in both groups (p>0.05). No significant differences were found for the 2 groups in median age (8 years), sex distribution (51% male), or the median number of students per school (635 [interquartile range 394–978]).

One-week baseline data were collected for intervention and control schools 2 weeks before the hand hygiene intervention activities. This step was carried out to test data collection procedures and ensure the collection of good quality data. No significant difference was found between the intervention group and control group in the rate of absenteeism caused by ILI (relative risk 1.1, 95% confidence interval [CI] 0.9–1.4) or in the rate of confirmed influenza episodes (relative risk 0.8, 95% CI 0.5–1.5).

During the 12-week observation period, 19,094 absences caused by overall illness were reported at the control schools (7.2 absences/100 student-weeks), compared with 13,247 absences in intervention schools (5.7 absences/100 student-weeks) (p<0.01). Across all schools, the overall reduction in absenteeism caused by illness was 21% in the intervention schools compared with the control (p<0.05). Absences caused by ILI, diarrhea, and conjunctivitis were reduced by 40%, 33%, and 67%, respectively, in the intervention group. No difference was observed for in-class reported illnesses between intervention and control schools; control and intervention schools reported 6,538 and 6,028 in-class illnesses, respectively (Table 2).

**Table 2:** Incidence of absences caused by illness and reasons for absence in control and intervention schools, Cairo, Egypt, February 16–May 12, 2008*

Absence caused by illness	Control, n = 282,832 student-weeks			Intervention, n = 250,584 student-weeks		Reduction, %	p value
	No. episodes	Median (IQR)		No. episodes	Median (IQR)		
Overall illness	19,094	7.2 (3.3–9.5)		13,247	5.7 (3.4–7.6)	21	<0.0001
ILI	1,671	0.5 (0.3–1.1)		917	0.3 (0.1–0.7)	40	<0.0001
Diarrhea	1,316	0.3 (0.1–0.6)		639	0.2 (0.0–0.5)	33	<0.0001
Conjunctivitis	1,214	0.3 (0.1–0.6)		530	0.1 (0.0–0.4)	67	<0.0001

*IQR, interquartile range; ILI, influenza-like illness.

**Figure 1:**
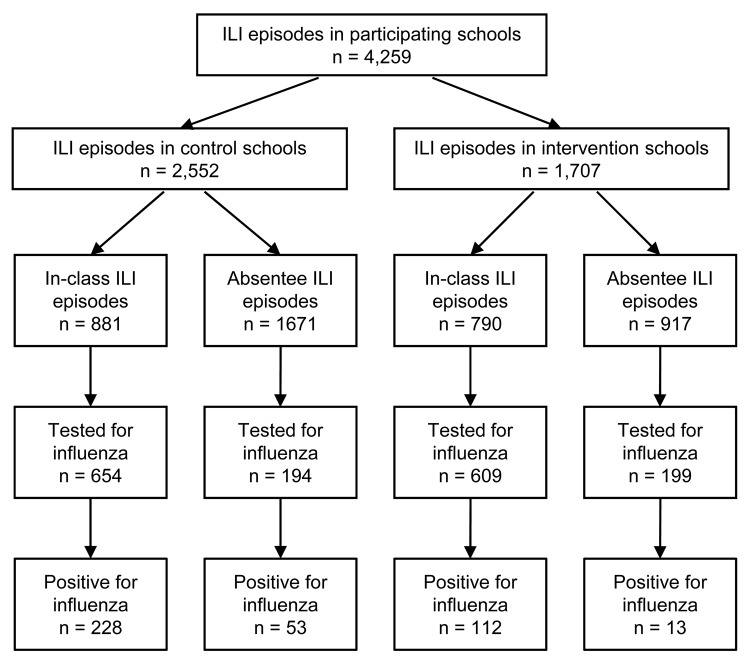
Episodes of absence because of influenza-like illness, diarrhea, and conjunctivitis in the intervention and control schools, by weeks, Cairo, Egypt, February–May 2008. Error bars indicate SEM.

Incidence of absence (per 100 students) caused by ILI was lower in the intervention group than in the control group for weeks 1–4, 5–8, and 9–12. During the first 4-week period, the 2 groups showed no differences in absence incidence caused by diarrhea and conjunctivitis, although the incidence of absence caused by these conditions was significantly lower in the intervention group for weeks 5–8 and weeks 9–12 (Figure 1).

**Figure 2:**
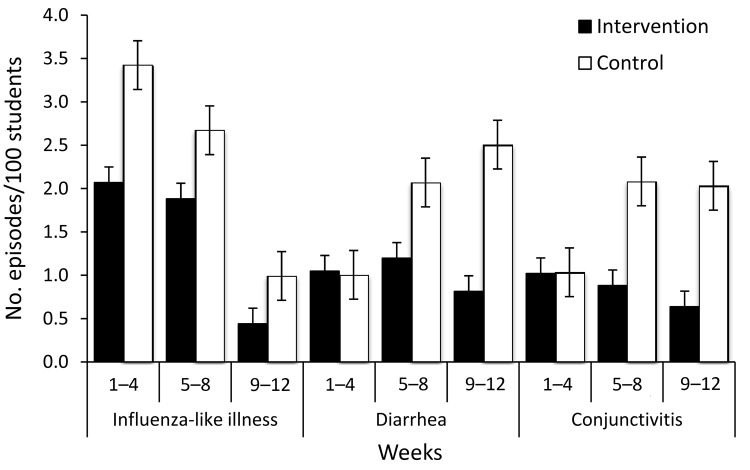
Diagram of results of influenza testing for students with influenza-like illness (ILI) in intervention and control schools, Cairo, Egypt, February–May 2008. Testing was done with QuickVue Rapid Antigen Test (Quidel Corp., San Diego, CA, USA).

During the 12-week observation period, 4,259 students were diagnosed with ILI in the control and intervention schools (n = 2,552 and n = 1,707, respectively). In the control schools, 881 cases (34.5%) of ILI were identified through in-class reported illness and 74.2% of these students (n = 654) were tested for influenza, of which 34.9% (n = 228) were positive. In contrast, 790 cases (46.3%) of ILI in the intervention schools were identified through in-class reporting, and while a similar proportion was tested for influenza virus (77%) (n = 609), only 18.4% (n = 112) were positive for influenza virus (p<0.01). In control schools, 65.5% (n = 1,671) of students were absent caused by ILI, of which 11.6% (n = 194) were tested and 27.3% (n = 53) were positive for influenza virus. In the intervention schools, ILI was responsible for 53.7% (n = 917) of absenteeism. Of these 917 students, 199 (21.7%) were tested and only 6.5% (n = 13) were positive for influenza virus (p<0.01) (Figure 2).

**Figure 3:**
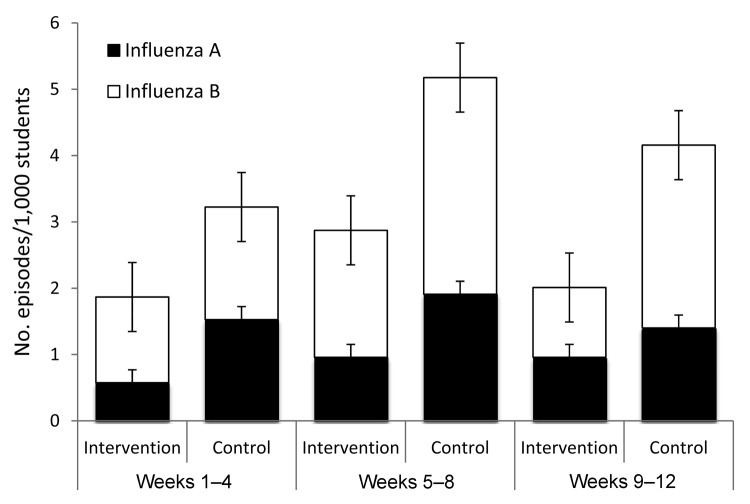
Episodes of laboratory-confirmed influenza A and B in the intervention and control schools, by weeks, Cairo, Egypt, February–May 2008. Error bars indicate SEM.

The incidence of laboratory-confirmed influenza (A and B) per 1,000 students was significantly lower in the intervention group than in the control group over the 12-week observation period as well as during weeks 1–4 (p<0.01) 5–8 (p<0.001), and 9–12 (p<0.001) (Figure 3). The incidence of influenza A in the intervention group was significantly lower during weeks 1–4 and 5–8, with no statistical difference in weeks 9–12. The incidence of influenza B was significantly lower in the intervention group during weeks 5–8 and 9–12.

The largest number of confirmed cases reported from any single school was from a control school where 66 cases were reported (7.24 cases/1,000 student-weeks). Overall, control schools were 1.6× (95% CI 1.1–2.2) more likely to report at least 1 confirmed case of influenza and 2.8× (95% CI 1.7–4.7) more likely to report multiple confirmed cases of influenza than intervention schools.

The monitoring teams observed during their regular visits that ≈93% of the students in the intervention schools had soap and drying material available. All intervention schools (except 2) had a rigorous system of ensuring that schoolchildren were washing their hands at least twice daily.

## Discussion

Elementary schoolchildren are important vectors for spreading infectious diseases between themselves, their families, and their communities, especially in developing countries where public schools are extremely overcrowded. Aiello et al. noted that infectious agents that children contract in schools can result in infections in up to 50% of household members (*17*).

Influenza transmission dynamics and potential methods for control are of particular interest in Egypt where avian influenza (H5N1) is endemic in poultry facilities, both commercial and backyard (*18*). Concern persists regarding the potential for recombination between seasonal influenza and subtype H5N1 strains with resultant rapid transmission of the recombinant strain, especially among high-density populations such as public school students and staff. In addition, nonpharmaceutical interventions, especially hand hygiene to mitigate pandemic (H1N1) 2009 disease spread, have been advocated by international organizations (*19*).

This randomized controlled intervention trial replicates well-known findings that intensive hand hygiene campaigns are highly successful in reducing absenteeism caused by illness and absenteeism caused by to ILI and diarrheal diseases among schoolchildren (*16*,*20*–*22*). This study also duplicates recent findings that incidence of in-class reported illness is not significantly decreased by promotion of hand hygiene (*16*). However, this study also demonstrates a decrease in laboratory-confirmed influenza as a result of an intensive hand hygiene campaign.

The 21% reduction in absenteeism caused by illness in intervention schools in this study is lower than the 42% observed among schoolchildren in the People’s Republic of China (*16*) and in similar US studies (*20*,*21*). These differences might be caused by the nature of the interventions implemented: ensuring a continuous free supply of soap as in China (*16*), promoting the use of alcohol-free instant hand sanitizers (*20*), or equipping classrooms with dispensers containing alcohol as described by Guinan et al. (*21*).

In this 12-week hand hygiene trial, absenteeism caused by ILI decreased 40% and laboratory-confirmed influenza decreased 47% in intervention schools relative to control schools. These reductions are higher than the 21% and 16% reductions in respiratory illness reported in 2 meta-analyses of hand hygiene interventions in community settings (*22*,*23*) and the elementary school based hand hygiene program in China (*16*). A recent study evaluating the effectiveness of hand hygiene and facemasks in preventing influenza transmission in households in Hong Kong showed reduction in influenza transmission, but the differences were not significant (*24*). Differences in study design (household-based versus school-based) or in intensity of the intervention may have contributed to the positive effects in our study. Also, the greater relative reductions of influenza in our study might be attributed to specific influenza transmission dynamics for Egypt or the season when the study was conducted. In addition, Egypt’s unique hand hygiene campaign required students to wash hands at least twice during the school day, which might have had a direct influence on reducing influenza.

The incidence of absenteeism caused by diarrhea was 33% lower in schoolchildren in the intervention schools. This result is similar to a Cochrane Reviews report that handwashing reduced the incidence of diarrheal episodes in children and adults by 30% (*11*). However, our study found a greater reduction in diarrhea than did a controlled trial conducted at a single elementary school (*8*), where the intervention only focused on providing alcohol-based hand sanitizers and wipes to disinfect classroom surfaces. Higher reduction rates of absenteeism caused by diarrhea (47%) were described in community settings that used soap for handwashing (*25*).

Notably, the incidence of ILIs decreased more than did diarrheal disease (40% vs 33%) in this study. Previous studies have shown a greater reduction in diarrheal disease incidence, possibly because it is easier to adopt handwashing behaviors associated with diarrhea such as preparing/eating food, defecating, etc., relative to those associated with ILI such as washing hands after sneezing or coughing. Also of interest in this study was the marked (67%) decrease in absenteeism caused by conjunctivitis in intervention schools compared to results for control schools.

There are several important limitations in this study. First, because study teams and schoolchildren and their parents were not blinded to the intervention, underreporting of illness as a cause for absenteeism in the intervention schools may have contributed to information bias. However, a rigorous system for identifying the reasons for illness based on a standardized list of symptoms as well as regular monitoring visits did not uncover any systematic errors. Differential interest of study teams may have contributed to the low rate of testing in students who were absent because of ILI in the control schools compared to the intervention schools (12% vs 22%); however, because nasal swabs were collected only from students who returned to school within 3 days of illness onset, it is unlikely that samples tested reflected the most severe manifestation of illness. Absence incidence, defined as >1 day of absence in given week, may have been overestimated if a child were ill at the end of 1 week and at the beginning of the subsequent week; however, such overestimation is unlikely to have occurred differentially between the intervention and control schools.

The relatively short duration of observation (12 weeks) may have also led to an overestimation of effect, as participants may have been more likely to adhere to new hand hygiene behavior over a shorter period. This study was not designed to measure sustainability of effect. In addition, the use of rapid tests for diagnosis of laboratory-confirmed influenza with known low sensitivity (60% in some studies) likely resulted in an underestimation of illness in each group; this would likely bias the effects towards the null. Finally, because of delays in scientific and official approvals, the campaign was not started until the end of the influenza season; a higher baseline prevalence of respiratory and diarrheal diseases during the trial period may have led to a stronger program effect on disease-specific absenteeism.

This intensive hand hygiene intervention was effective in reducing transmission of influenza among schoolchildren and was feasible and acceptable. In spite of operational difficulties in schools, the Egyptian Ministry of Health recommended hand hygiene as a means of reducing transmission of pandemic (H1N1) 2009 and other infectious diseases on a countrywide basis by using mass media campaigns (television and radio programs) and focusing on schoolchildren. Sustaining intensive national hand hygiene programs is challenging because of the high costs of training, printing materials, and logistics involved. In addition, the lack of continuous availability of soap and water is a limiting factor in remote areas. Identifying strategies that provide national, long-term, cost-effective alternatives to promote hand hygiene is critical in preventing transmission of diarrheal diseases and emerging respiratory viruses of pandemic potential.
